# RETRACTION: Aerial imagery reveals abnormal stingrays, *Taeniura lymma* (Myliobatiformes: Dasyatidae), in the central Red Sea

**DOI:** 10.1002/ece3.70246

**Published:** 2024-09-04

## Abstract

**RETRACTION:** I.A. Ciocănaru, B.O. Nieuwenhuis, R.L. Ostrovski, J. Cochran, and B.H. Jones, “Aerial Imagery Reveals Abnormal Stingrays, Taeniura Lymma (Myliobatiformes: Dasyatidae), in the Central Red Sea,” *Ecology and Evolution* 14, no. 6 (2024): e11399, https://doi.org/10.1002/ece3.11399.

The above article, published online on 30 May 2024 in Wiley Online Library (wileyonlinelibrary.com), has been retracted by agreement between the journal Editor‐in‐Chief, Arley Muth, and John Wiley & Sons, Ltd. The retraction has been agreed due to an error in the differentiation of specimens in Figure 1. As a result, the conclusions reported in the article are not considered reliable. A revised version of this article is forthcoming. The corresponding author Ioana Andreea Ciocănaru agrees with this decision on behalf of all authors.

**RETRACTION:** I.A. Ciocănaru, B.O. Nieuwenhuis, R.L. Ostrovski, J. Cochran, and B.H. Jones, “Aerial Imagery Reveals Abnormal Stingrays, Taeniura Lymma (Myliobatiformes: Dasyatidae), in the Central Red Sea,” *Ecology and Evolution* 14, no. 6 (2024): e11399, https://doi.org/10.1002/ece3.11399.

The above article, published online on 30 May 2024 in Wiley Online Library (wileyonlinelibrary.com), has been retracted by agreement between the journal Editor‐in‐Chief, Arley Muth, and John Wiley & Sons, Ltd. The retraction has been agreed due to an error in the differentiation of specimens in Figure 1. As a result, the conclusions reported in the article are not considered reliable. A revised version of this article is forthcoming. The corresponding author Ioana Andreea Ciocănaru agrees with this decision on behalf of all authors.

